# Prevalence of human papillomavirus in the cervical epithelium of Mexican women: meta-analysis

**DOI:** 10.1186/1750-9378-7-34

**Published:** 2012-12-03

**Authors:** Raúl Peralta-Rodríguez, Pablo Romero-Morelos, Vanessa Villegas-Ruíz, Mónica Mendoza-Rodríguez, Keiko Taniguchi-Ponciano, Beatriz González-Yebra, Daniel Marrero-Rodríguez, Mauricio Salcedo

**Affiliations:** 1 Laboratorio de Oncología Genómica, Unidad de Investigación Médica en Enfermedades Oncológicas, Hospital de Oncología, CMN SXXI-IMSS, Av. Cuauhtémoc 330, Col. Doctores, México, Distrito Federal 06720, México; 2 Departamento de Medicina y Nutrición, División de Ciencias de la Salud, Campus León, Universidad de Guanajuato, Dept. Investigacion, Hospital Regional de Alta Especialidad del Bajio, Leon, Guanajuato, México; 3 Laboratorio de Biotecnología, Universidad Autonóma de Ciudad Juárez, Cd. Juárez, Chihuahua, México

**Keywords:** Meta-analysis, Cervical carcinoma, HPV

## Abstract

**Background:**

Human Papillomavirus (HPV) in cervical epithelium has been identified as the main etiological factor in the developing of Cervical Cancer (CC), which has recently become a public health problem in Mexico. This finding has allowed for the development of vaccines that help prevent this infection. In the present study, we aimed to determine the prevalence and HPV type-distribution in Mexican women with CC, high-grade squamous intraepithelial lesion (HSIL), low-grade squamous intraepithelial lesion (LSIL), and Normal cytology (N) to estimate the impact of the HPV vaccines.

**Methods:**

The PubMed database was used to identify and review all articles that reported data on HPV prevalence in CC, precursor lesions, and normal cytology of Mexican women.

**Results:**

A total of 8,706 samples of the tissues of Mexican women were stratified according to diagnosis as follows: 499 for CC; 364 for HSIL; 1,425 for LSIL, and 6,418 for N. According to the results, the most prevalent genotypes are the following: HPV16 (63.1%), -18 (8.6%), -58, and −31 (5%) for CC; HPV-16 (28.3%), 58 (12.6%), 18 (7.4%), and 33 (6.5%) for HSIL; HPV-16 (13.1%), 33 (7.4%), 18 (4.2%), and 58 (2.6%) for LSIL, and HPV-16 (3.4%), 33 (2.1%), 18, and 58 (1.2%) for N.

**Conclusions:**

Taken together, genotypes 58 and 31 (10%) are more common than type 18 (8.6%) in CC. Therefore, the inclusion of these two genotypes in a second-generation vaccine would provide optimal prevention of CC in Mexico.

## Background

Cervical cancer (CC) is one of the main causes of death in women in developing countries [1]. In 2008, an estimate of 10,186 Mexican women developed Cervical Cancer (CC) and 5,061 died as a result of this disease [2]. The role of the Human papillomavirus (HPV) in the etiology of CC precursor lesions and invasive carcinoma development has been well established. Persistent infection with High-Risk HPV (HR-HPV) types has been recognized as a factor for CC development [3]. Highlighting the HR-HPV types 16 and 18 are associated with over 70% of CC worldwide [4] being the most frequent HPV16.

There are previous meta-analysis papers, which report information on the HPV-type prevalence distribution in precursor lesions and CC worldwide [5-8]. However, these data are variable and incomplete for Mexican population. A recent meta-analysis that included 115,789 HPV-positive female samples of precursor lesions and CC, HPV types 16, 18, and 45 presented different prevalence rates, and they comprised the most common viral types in CC worldwide [7]. The HPV16 type is also the most common genotype in normal cytological samples from healthy women worldwide [8]. Regional data on type-distribution is essential for estimating the impact of vaccines on CC and for the development of screening programs.

In order to have a possible HPV status in Mexico, the most complete of the already published data on cervical lesions and normal cytology were reviewed.

## Results

### Review of the HPV prevalence

**Figure 1:**
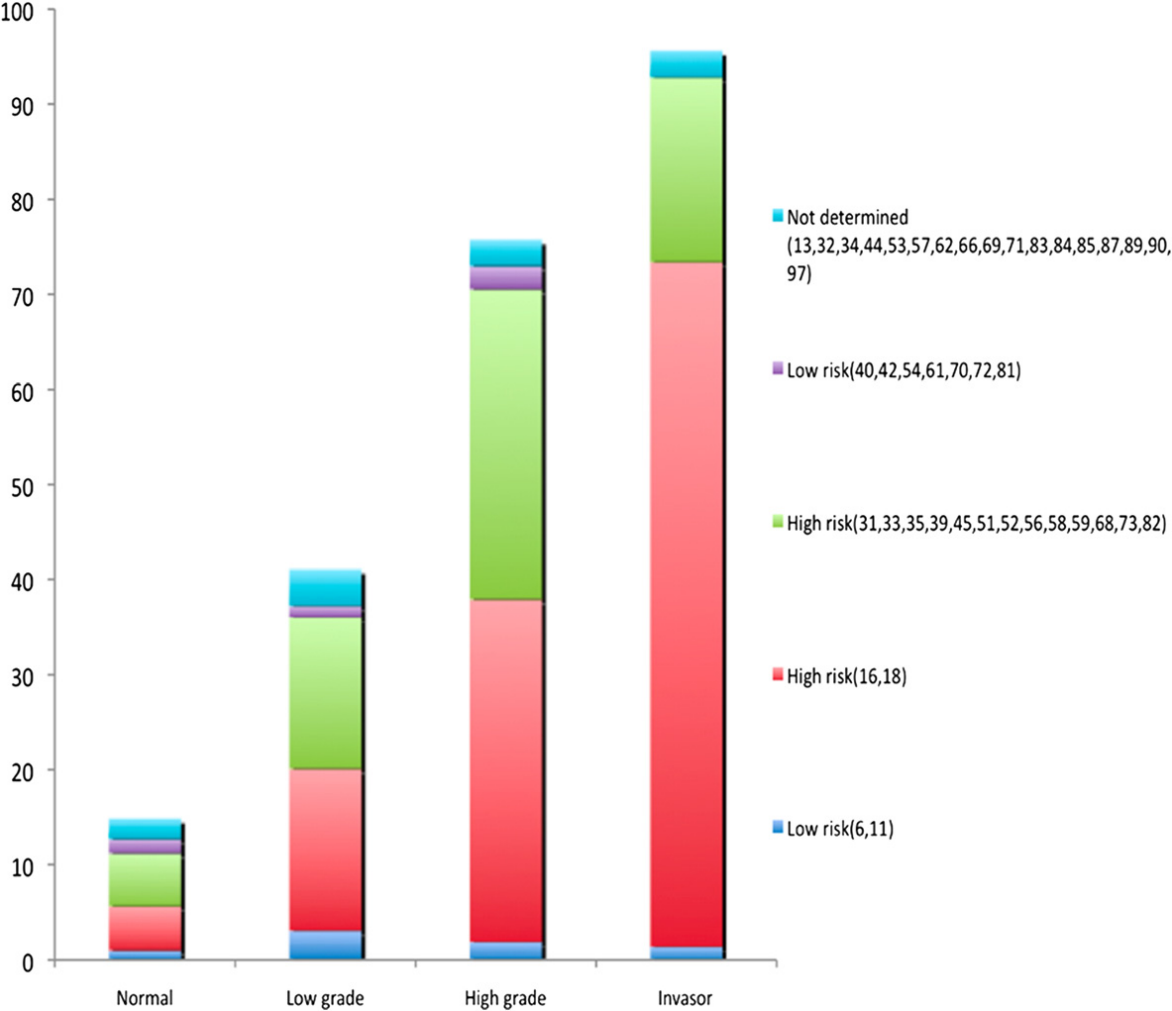
**HPV prevalence in Mexican women. **The HPV prevalence is expressed as percentage of all cases submitted to molecular detection of HPV. From 8,706 samples of Mexican women, 476/499 were positive for HPV and CC, 275/364 were positive for HPV and HSIL, 601/1,425 were positive for HPV and LSIL, 967/6,418 were positive for HPV and cytology normal.

**Figure 2:**
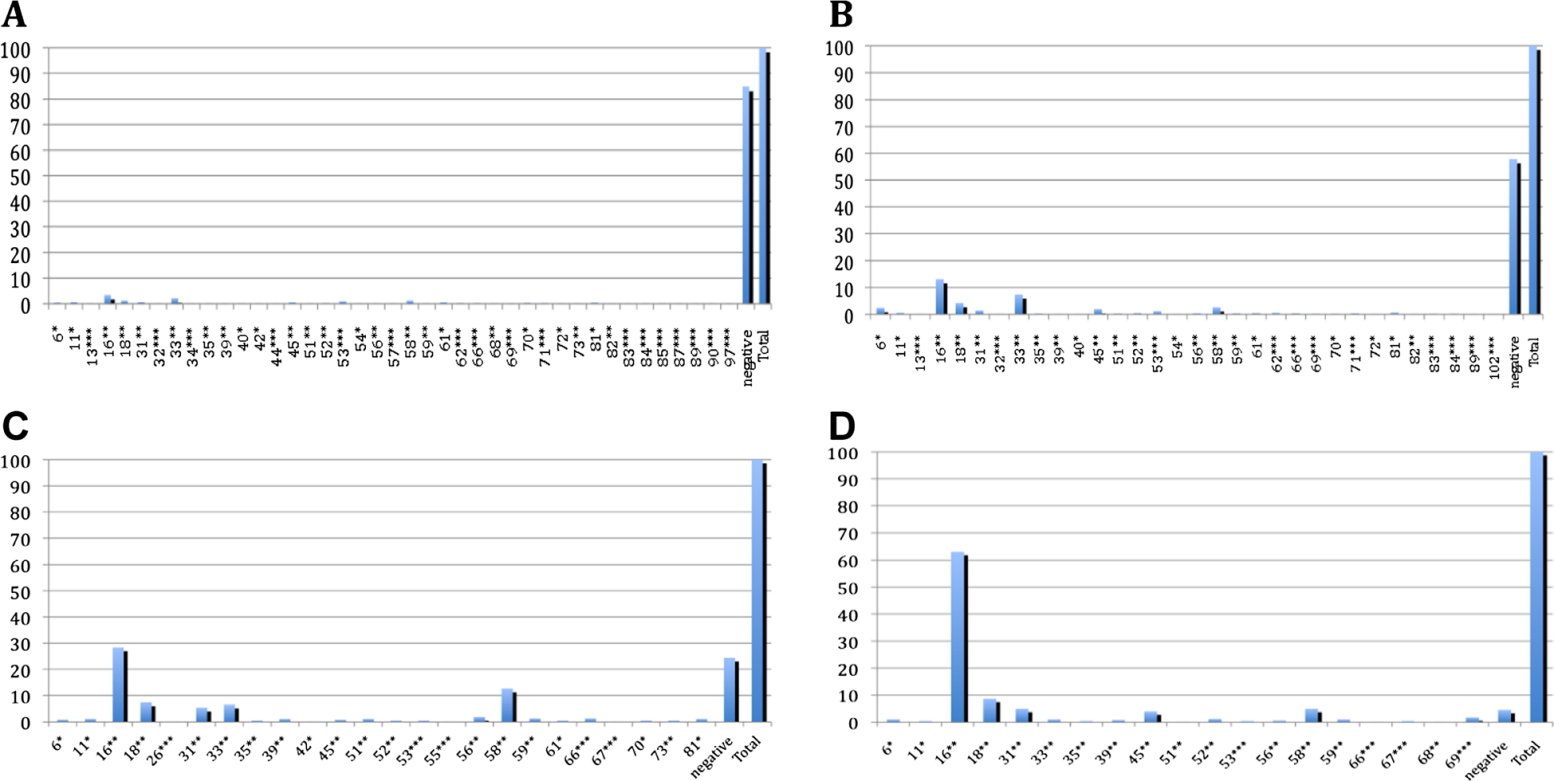
**A. HPV prevalence in women with normal cytology. B. **HPV prevalence in women with LSIL. **C. **HPV prevalence in women with HSIL. **D. **HPV prevalence in women with CC. *: Low risk HPV (no oncogenic potential); **: high risk HPV (oncogenic potential); ***: HPV without sufficient epidemiological evidence to know their risk of oncogenesis (2).

In the present report, we analyzed the HPV prevalence from 12 already published reports that met the aforementioned criteria. There were a total of 8,706 cases, of which 499 were identified as CC, 364 as HSIL, 1,425 as LSIL, and 6,418 as N. Overall, HPV prevalence was 95, 75.5, 42, and 15%, respectively (Figure 1). In CC, HPV-16 (63.1%), -18 (8.6%), -58, and −31 (5%) were the most prevalent types, while for HSIL, these were HPV-16 (28.3%), -58 (12.6%), -18 (7.4%), and −33 (6.5%), for LSIL, HPV-16 (13.1%), -33 (7.4%), -18 (4.2%), and −58 (2.6%) and for cases with normal cytology, these were HPV-16 (3.4%), -33 (2.1%), -58 and −18 (1.2%) (Table 1) (Figure 2).

**Table 1:** Overall prevalence of HPV types found in Mexican women according to type of lesion and normal cytology

**Type**	**CC**		**HSIL**		**LSIL**		**Normal**		**All**	
**HPV**	**n**	**%**	**N**	**%**	**N**	**%**	**N**	**%**	**N**	**%**
6*	5	1.00	3	0.82	35	2.46	26	0.41	69	0.79
11*	2	0.40	4	1.10	9	0.63	39	0.61	54	0.62
13***	0	0.00	0	0.00	1	0.07	1	0.02	2	0.02
16**	315	63.13	103	28.30	188	13.19	221	3.44	827	9.50
18**	43	8.62	27	7.42	61	4.28	78	1.22	209	2.40
26***	0	0.00	1	0.27	0	0.00	0	0.00	1	0.01
31**	25	5.01	20	5.49	21	1.47	39	0.61	105	1.21
32***	0	0.00	0	0.00	2	0.14	4	0.06	6	0.07
33**	5	1.00	24	6.59	106	7.44	138	2.15	273	3.14
34***	0	0.00	0	0.00	0	0.00	2	0.03	2	0.02
35**	2	0.40	2	0.55	5	0.35	12	0.19	21	0.24
39**	4	0.80	4	1.10	2	0.14	12	0.19	22	0.25
40*	0	0.00	0	0.00	1	0.07	5	0.08	6	0.07
42*	0	0.00	1	0.27	0	0.00	1	0.02	2	0.02
44***	0	0.00	0	0.00	0	0.00	1	0.02	1	0.01
45**	20	4.01	3	0.82	28	1.96	32	0.50	83	0.95
51**	1	0.20	4	1.10	5	0.35	12	0.19	22	0.25
52**	6	1.20	2	0.55	8	0.56	14	0.22	30	0.34
53***	2	0.40	2	0.55	16	1.12	59	0.92	79	0.91
54*	0	0.00	0	0.00	2	0.14	5	0.08	7	0.08
55***	0	0.00	1	0.27	0	0.00	0	0.00	1	0.01
56**	3	0.60	7	1.92	6	0.42	3	0.05	19	0.22
57***	0	0.00	0	0.00	0	0.00	2	0.03	2	0.02
58**	25	5.01	46	12.64	38	2.67	79	1.23	188	2.16
59**	5	1.00	5	1.37	7	0.49	12	0.19	29	0.33
61*	0	0.00	2	0.55	8	0.56	33	0.51	43	0.49
62***	0	0.00	0	0.00	9	0.63	15	0.23	24	0.28
66***	1	0.20	5	1.37	7	0.49	13	0.20	26	0.30
67***	2	0.40	1	0.27	0	0.00	0	0.00	3	0.03
68**	1	0.20	0	0.00	0	0.00	8	0.12	9	0.10
69***	9	1.80	0	0.00	4	0.28	6	0.09	19	0.22
70*	0	0.00	2	0.55	4	0.28	24	0.37	30	0.34
71***	0	0.00	0	0.00	6	0.42	17	0.26	23	0.26
72*	0	0.00	0	0.00	1	0.07	3	0.05	4	0.05
73**	0	0.00	2	0.55	0	0.00	1	0.02	3	0.03
81*	0	0.00	4	1.10	10	0.70	28	0.44	42	0.48
82**	0	0.00	0	0.00	1	0.07	1	0.02	2	0.02
83***	0	0.00	0	0.00	4	0.28	6	0.09	10	0.11
84***	0	0.00	0	0.00	4	0.28	2	0.03	6	0.07
85***	0	0.00	0	0.00	0	0.00	3	0.05	3	0.03
87***	0	0.00	0	0.00	0	0.00	1	0.02	1	0.01
89***	0	0.00	0	0.00	1	0.07	4	0.06	5	0.06
90***	0	0.00	0	0.00	0	0.00	4	0.06	4	0.05
97***	0	0.00	0	0.00	0	0.00	1	0.02	1	0.01
102***	0	0.00	0	0.00	1	0.07	0	0.00	1	0.01
negatives	23	4.61	89	24.45	824	57.82	5,451	84.93	6,387	73.36
**Total**	**499**	**100.00**	**364**	**100.00**	**1,425**	**100.00**	**6,418**	**100.00**	**8,706**	**100.00**

*n *= number of samples; %: frequency; *CC*: cervical carcinoma, *HSIL*: high-grade squamous intraepithelial lesion, *LSIL*: low grade squamous intraepithelial lesion, Normal: Women without lesion and normal cytology. *: HPV Low risk (no oncogenic potential); **: HPV high risk (oncogenic potential); ***: HPV without sufficient epidemiological evidence to know their risk of oncogenesis [2].

**Figure 3:**
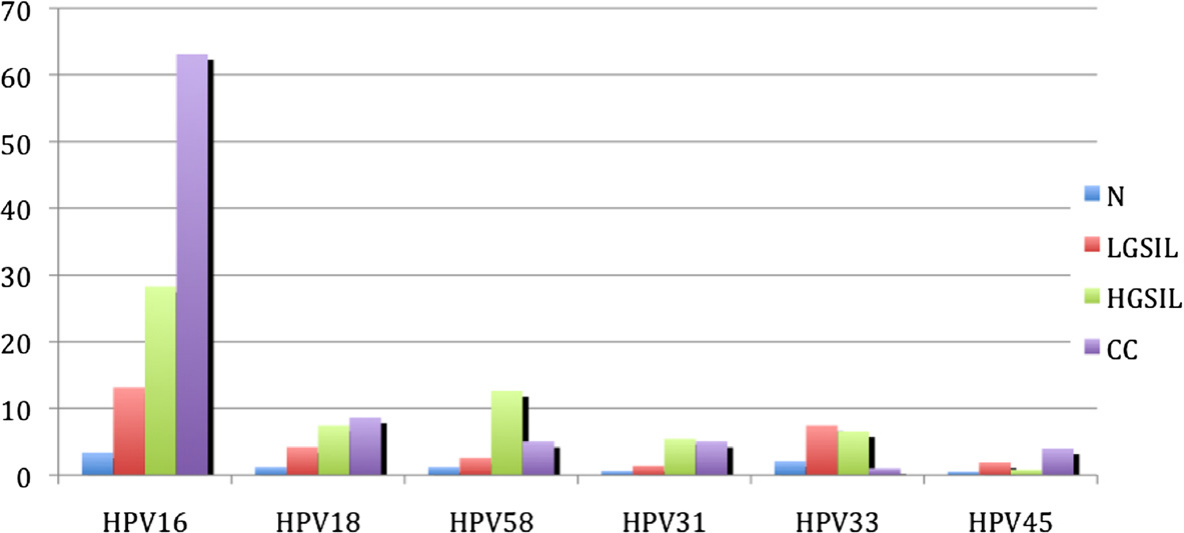
**Comparison of the most common HPV genotypes in each type of cervical lesion and normal cytology. **HPV prevalence (types 16, 18, 58, 31, 33 and 45) in each type of cervical lesion and normal cytology. The genotypes analyzed correspond to the high risk and are most frequently found in cervical epithelium.

HPV-16 was the most prevalent type in each cervical lesion and in normal cytology (Figure 3). In summary, the HPV-16/18 fraction was the most prevalent in CC, while in HSIL, the HPV-16/58 fraction was the most prevalent; for both low-grade lesions and normal tissue, the HPV-16/33 fraction was the most prevalent (Table 1).

In CC, there were variations in HPV type prevalence in the Western, Central and South regions of Mexico. In the West region, HPV-16 and −58 were followed by −18 and −33. In the Central region, the same behavior was observed on HPV types −16, -18, -45, and −58, while the South region shows prevalence on HPV types −16, -18, -31, and −58 (Table 2). There was no variation in HPV prevalence for HSIL cases in the West and Central regions, where HPV-16, -58, and −18, were found, while types −16, -33, and −58 were found in the South region. In N cases, findings show the most drastic variation in HPV prevalence of the three regions. The West region shows HPV prevalence for types −16, -58, and −18; in the Central region, for types −16, -18, and 31; and in the South for types −16, -33, and −58 (Table 2).

**Table 2:** Regional distribution of HPV infection in positive woman

**Region**	**West**	**Central**						**South**			
**n**	**n (141)**	**n (18)**	**n (11)**	**n (187)**	**n (183)**	**n (94)**	**n (161)**	**n (617)**	**n (418)**	**n (162)**	**n (304)**
**HPV type**	**N (%)**	**HSIL (%)**	**CC (%)**	**N (%)**	**LSIL (%)**	**HSIL (%)**	**CC (%)**	**N (%)**	**LSIL (%)**	**HSIL (%)**	**CC (%)**
16	24.1	50	72.7	17.1	50.8	48.9	57.2	22.2	22.7	29.6	65.7
18	7.1	5.6	9.1	17.1	25.1	12.7	8	5.5	3.5	8.6	8.8
31	4.3	0	0	6.9	4.3	6.3	2.1	3.2	3.1	14	6.9
33	5.7	0	0	5.3	0	3.1	0.5	19.4	25.3	21	1.3
45	5.7	0	0	2.1	2.7	1	4.3	3.2	5.5	1.2	3.9
58	12.8	33.3	18.2	6.9	2.7	14.8	2.7	7.8	7.8	16	5.9

HPV prevalence percentages in three regions of Mexico (west, central and south region).

The differences in HPV prevalence for each group (CC, HSIL, LSIL, and normal) were assessed by contingency tables and the *x*^2^ test. The most prevalent genotypes in each type of cervical lesion and normal cytology were statistically associated with the pathological diagnosis (*p* <0.05) (Table 3). The difference in the prevalence of HPV16, -33, -45, and 58 between CC and HSIL was very significant (*p* = 0.000), while in HPV18 and 31 it was not (*p* >0.05) (Table 3). The statistical difference for HPV16, -18, -31, -33, -45, and −58 between CC and LSIL was significant. (*p* <0.05) (Table 3). The difference of HPV16, -18, -31, -45, and −58 between CC and normal cytology was statistically significant (*p* <0.05), but in HPV33, the difference was not statistically significant (*p* >0.05) (Table 3).

**Table 3:** HPV prevalence analysis in CC, HGSIL, LGSIL and normal cytology

**Type**	**CC (%) n=499**	**HSIL (%) n=364**	**CC/HSIL PR (RR; 95% CI)**	**p**	**LSIL (%) n=1,425**	**CC/LSIL PR (RR; 95% CI)**	**p**	**Normal (%) n=6,418**	**CC/Normal PR (RR; 95% CI)**	**p**
Overall	476 (95.3)	275 (75.5)	1.2 (6.7; 1.86-3.88)	0.000	601 (42.1)	2.2 (28.3; 1.78-4.04)	0.000	997 (15.5)	6.1 (113.5; 1.77-4)	0.000
16	315 (63.1)	103 (28.3)	2.2 (4.3; 2.1-3.44)	0.000	188 (13.1)	4.8 (11.9; 2.30-3.13)	0.000	221 (3.4)	18.5 (49.1; 2.28-3.16)	0.000
18	78 (1.2)	27 (7.4)	0.1 (1.1; 2.21-3.27)	0.522	61 (4.2)	0.2 (2.1; 2.2-3.28)	0.000	78 (1.2)	1 (7.7; 2.08-3.46)	0.000
31	25 (5)	20 (5.4)	0.9 (0.9; 2.05-3.51)	0.752	21 (1.4)	3.5 (3.5; 2.03-3.56)	0.000	39 (0.6)	8.3 (8.6; 1.95-3.7)	0.000
33	5 (1)	24 (6.5)	0.1 (0.1; 1.21-5.98)	0.000	106 (7.4)	0.1 (0.1; 1.14-6.34)	0.000	138 (2.1)	0.4 (0.4; 1.13-6.38)	0.083
45	20 (4)	3 (0.8)	5 (5; 2.26-3.18)	0.004	28 (1.9)	2.1 (2; 1.9-3.8)	0.012	32 (0.5)	8 (8.3; 1.89-3.82)	0.000
58	25 (5)	46 (12.6)	0.3 (0.3; 1.95-3.7)	0.000	38 (2.6)	1.9 (1.9; 1.96-9.7)	0.011	79 (1.2)	4.1 (4.2; 1.89-3.81)	0.000

Differences in prevalence of HPV16, 18, 31, 33, 45 and 58 in each type of cervical lesion and normal cytology. The genotypes analyzed correspond to the high risk and are most frequently found in cervical epithelium. Differences were found between CC/HSIL, CC/LSIL and CC/normal cytology. Brackets shows the percentage.

The relative risk (RR; 95%) for HPV-16 infection between CC and HSIL was 4.3, and increased between CC and LSIL (11.9) and between CC and normal (49.1) (Table 3). The RR of HPV-18 infection between CC and HSIL was 1.1 and increased between CC and LSIL (2.1) and between CC and normal (7.7) (Table 3). The RR of HPV-31 infection between CC and HSIL was 0.9, and increased between CC and LSIL (3.5) and between CC and normal (8.6) (Table 3). RR of HPV-58 infection relative risk between CC and HSIL was 0.3, and also increased between CC and LSIL (1.9) and between CC and normal (4.2) (Table 3). RR of HPV-33 infection between CC and HSIL and LSIL is balanced (0.1), but increased slightly between CC and normal (0.4) (Table 3). RR of HPV-45 infection between CC and HSIL was 5 and decreased between CC and LSIL (2), but increased between CC and normal (8.3) (Table 3). In summary, the RR of HPV-16, -18, -31, and −58 infection increased between CC and precursor lesions and normal cytology, but HPV-33 and −45 did not increase between CC and precursor lesions and normal cytology (Table 3).

## Discussion

The development of cervical cancer is associated with the oncogenic HPV infection. These viruses contain early activity genes in its genome (*E1*, *E2*, *E4*, *E5*, *E6*, and *E7*) and late activity genes (*L1*, *L2*). The molecular evidence indicates that the oncogenic HPV proteins E6 and E7 are responsible for the inactivation of certain cell cycle regulatory proteins, such as p53 and pRb, respectively. The uncontrolled proliferative cellular activity that characterizes cells transformed by oncogenic HPV infection in CC and other cancers associated with this infection is due to the loss-of-function of proteins that arrest the cell cycle. Thus, this infection with oncogenic HPV results must play a central role in the developmental model of the CC [1].

The present meta-analysis helped to know the HPV prevalence and type-distribution in several regions of Mexico. From the total of the published reports, only 12 were included in the final meta-analysis, representing the 23.5% of the reports on HPV in Mexico [9-20]. In CC, the most common HPV types found were both HPV-16 and −18 (71.7%), with a similar prevalence to that reported worldwide. However, when performing a regional analysis, differences in the prevalence of this fraction in three regions (Central, South, and West) of Mexico were found. While in the Central and South regions, the HPV-16/18 fraction is the most prevalent, in the West region, the HPV-16/58 fraction is the most prevalent, with a percentage of 90.3 (Table 2). Thus, in this region there is a different HPV prevalence than that of reports worldwide. It is noteworthy that in women with high-grade lesions, HPV-58 is found as the second most prevalent (8.8%), followed by HPV18 (6.5%) and −31 (2.5%). The case of HPV-58 is quite similar to that of women of Southeast Asia (China, Japan) [21,22]. We hypothesized that there is a probability of finding genetic components that influence the infection by means of an “Asian HPV type” in Mexican population. In summary, with respect to the HPV-58 infection, we can mention that this HPV is common in Mexican population, its distribution is delimited to certain geographical regions (southern Mexico) and, by their high frequency in normal smears and precursor lesions, its infection possesses a high probability of leading to the progression of invasive lesions. Thus, there are necessary additional studies that might explain the biological differences between HPV-16 and −58 infections and the progression of cervical cancer.

Recently, an HPV immunization scheme was introduced in some countries, including Mexico. The strategy proposed for Mexico includes primary prevention with HPV vaccination for girls aged between 9 and 16 years (prior to sexual initiation). However, in some regions of Mexico, other genotypes that are not considered within this scheme are detected with high frequency. For example, the HPV-31 type is the second most common one in the states of Guanajuato and San Luis Potosí [9] (West region), and genotype 58 is the second most frequent in Yucatan [10] (South region). The high prevalence of HR-HPV types not present in current vaccines could be an important factor to consider within the strategies and policies of CC prevention.

On the other hand, with this type of vaccines, the rate of CC mortality is expected to be reduced. In general, Mexico currently has a mortality rate by CC of 9.2, which is lower than that of the South region considered in this analysis (12.2) and higher than that reported in the Central region (8.0) (Mortality in Women, Mexican Ministry of Health 2010). In this analysis, we found the highest prevalence in the South region for HPV-16 and −58 (76.7%).

Further studies of HPV testing are necessary to ascertain the real impact of the current vaccines, primarily because of the different HPV types found.

## Conclusions

HPV prevalence in Mexican women is heterogeneous according to lesion type and region. Thus, while for CC the most common HPV types are HPV-16 and −18 in high-grade lesions, the HPV-16 and −58 types are more frequent. Finally, in low-grade lesions and normal cytology, the most common genotypes were HPV-16 and −33.

According to the present analysis, a potential new-generation vaccine directed against groups A7 and −9 papillomavirus would protect our population in approximately 90% of cases. This would provide optimal prevention of CC in Mexico (and in the Asian region). Furthermore, this approach could serve as a base to promote new screening methods directed toward the most frequent HPV types.

## Methods

### Inclusion criteria and study selection

The PubMed database (National Library of Medicine, Bethesda, MD, USA) was used to identify articles published up to 2012 that had the combination of medical terms with the heading “Human papillomavirus” or “HPV” and “cervical cancer” or “cervical neoplasia” or “cervical intraepithelial neoplasia” and “Mexico”. We selected and reviewed all articles that reported data on HPV prevalence in CC, precursor lesions, and normal cytology of Mexican women. The analysis was restricted to studies that have the following inclusion criteria: a) that the samples analyzed were of Mexican women (cared for at any national health system, including the Mexican Institute of Social Security (IMSS), the Mexican Institute of Social Security Services of State Workers (ISSSTE), the Mexican Ministry of Health (SSa), and the Mexican Ministry of National Defense (SEDENA); b) that the report contained a clear description of the pathology or cytology and classification determined as CC (squamous cell carcinoma and adenocarcinoma, taken as two separate entities); high-grade squamous intraepithelial lesion (HSIL) according to the Bethesda system, or Cervical intraepithelial neoplasia 2 or 3 (CIN2, CIN3); Low-grade squamous intraepithelial lesion (LSIL), or Cervical intraepithelial neoplasia 1 (CIN1) and normal cytology; c) that the report included at least one of the four classifications: CC; HSIL or CIN2/CIN3 alternatively, LSIL, or CIN1, normal, and that the number of samples included in the report was >10, and d) that the detection method comprised at least viral genotypes 16, 18, and some others, and that it would be conducted by 1) Polymerase chain reaction [PCR]+direct sequencing, or 2) PCR+restriction fragment length polymorphisms, or 3) PCR+reverse line blot.

A search of the literature identified 51 studies, of which only 12 could be included in the final meta-analysis. Exclusion criteria were the following: a) only HPV16 and −18 genotypes were studied; b) HPV type was not specified (hybrid capture); c) number of cases was carried out for <10; d) a method other than PCR was used to detect HPV types, e) the histological category was not reported as normal, LSIL, HSIL, or CC.

### Estimation of HPV prevalence

Overall, HPV prevalence was estimated by dividing the total number of subjects for each histological group by the number of HPV-positive cases. Type-specific HPV prevalence is presented for each histological classification for all HPV types identified. Multiple infections were not considered in this analysis.

### Statistical analysis

The differences in HPV prevalence for each group (CC, HSIL, LSIL, and normal) were assessed by contingency tables. The *x*^2^ test was conducted and considered of statistical significance with a level of *p* <0.05. Type-specific HPV prevalence was compared between HSIL/LSIL/normal and CC by Prevalence ratios (PR) and Relative risk (RR) with 95% Confidence intervals (95% CI). The analyses were performed by SPSS v15 software.

## Competing interest

The authors’ declare that they have no competing interests.

## Author’s contributions

RP, BG and MS conceived and designed the study, analyzed data and drafted the manuscript, PR, VV, MM, KT and DM helped with statistical analysis. All authors read and approved the final manuscript.
